# Reversible dilated cardiomyopathy caused by hypothyroidism

**DOI:** 10.1186/1755-7682-4-20

**Published:** 2011-06-21

**Authors:** I Khochtali, N Hamza, O Harzallah, S Hamdi, J Saad, M Golli, S Mahjoub

**Affiliations:** 1 Department of Internal Medicine, Endocrinology Unit. Monastir University Hospital 5000, Tunisia; 2 Department of Cardiology, Monastir University Hospital, Monastir 5000, Tunisia; 3 Department of Radiology, Monastir University Hospital, Monastir 5000, Tunisia

## Abstract

The association between lack of thyroid hormones and cardiac dysfunction has been well described. We report two new cases of patients with dilated cardiomyopathy (DCM), revealing a periphery hypothyroidism and for whom cardiac function significantly improved after L thyroxin substitutive treatment. Our cases highlight the necessity to perform thyroid function testing to investigate the etiology of non ischemic DCM.

## Introduction

Dilated cardiomyopathy (DCM) is a heart muscle disorder defined by the presence of a dilated and poorly functioning left ventricle in the absence of abnormal loading conditions (hypertension, valve disease) or ischemic heart disease sufficient to cause global systolic impairment [1]. In the majority of patients no identifiable cause is found hence the term "idiopathic" dilated cardiomyopathy (IDC). However, in a rare occasions DCM could be associated to variety of pathologies. A link between CMD and hypothyroidism was first reported in 1918 in four patients with heart failure refractory to digitalis and diuretics treated successfully with substitutive hormonal therapy [2]. Since this first description few other cases have been published.

We report, two cases of hypothyroidism revealed by a DCM.

## Observations

### First case

A 20 years old man, without any previous medical history, was admitted for chest pain and dyspnea. One year earlier, the patient developed an important muscle weakness and a weight gain. On examination the patient was obese, with a body mass index (BMI) at 35.4 kg/m^2, ^his skin was pale, dry and infiltrated with edema. Blood pressure was at 120/80 mmHg, the pulse was at 50 b/mn and heart sounds were distant. Chest X ray showed moderate cardiomegaly with a cardiothoracic ratio of 0.59. Cardiac ultrasound revealed a DCM with a severe left ventricle dysfunction (ejection fraction at 20%) and a global hypokinesis. In addition, there was a moderate pericardial effusion.

Biological tests showed high cholesterol and triglyceride level respectively at 6.72 mmol/l and 3.72 mmol/l, hyponatremia (132 mmol/l), elevated transaminases (SGOT 52 UI/l and SGPT 86 UI/l) and a raised creatinine phosphokinase level (475 UI/l). Thyroid hormone's dosage showed decrease of free thyroxine level (T4 = 0.72 ng/ml) and an elevation of thyroid stimulating hormone (TSH = 369.5 mUI/l) supporting peripheral origin of hypothyroidism. The thyroid gland was enlarged, heterogeneous and multinodular on ultrasound imaging. The anti-thyroperoxidase and anti-microsomial antibodies were positive. The final diagnosis was Hashimoto thyroiditis. The patient was treated with substitutive hormonal treatment with thyroxin at an initial dose of 25 μg/day and then progressively increased until a dosage of 150 μg/day. Three months after, cardiac ultrasound showed a significant improvement of the left ventricle systolic ejection fraction to 47% (Table 1).

**Table 1:** Biological tests and cardiac ultrasound results before and after substitutive treatment for 2 patients

	*Before substitutive treatment *	*After substitutive treatment (12 weeks)*	*Reference* *ranges *
TSH			
Patient 1	396,5	24	0,25 - 4 (mUI/l)
Patient 2	23.4	5	

Hemoglobin			
Patient 1	11.2	12.3	13-16 g/dl
Patient 2	13.1	13.2	

CPK			
Patient 1	496	100	< 250 (UI/l)
Patient 2	550	230	

Cholesterolemia			
Patient 1	6,72	5.8	< 5,5 (mmol/l)
Patient 2	7.1	5,7	

Ejection fraction			
Patient 1	20%	49%	55%
Patient 2	25%	47%	

TSH: thyroid stimulating hormone

CPK: creatin phosphokinase

### Second case

A 34 years old man was hospitalized to investigate his DCM recently discovered. His medical history was marked by diabetes and heavy smoking. Four months ago, the patient reported weight gain, constipation and a nocturnal snoring developed. The physical exam revealed a patient with a BMI of 31.86 kg/m^2^, a blood pressure at 120/80 mmHg and a mild palpable goiter.

Thyroid hormones dosage revealed a peripheral hypothyroidism (TSH = 23.4 mUI/l and T4 = 10 ng/ml). Cardiac ultra sound showed a decrease of the left ventricle ejection fraction at 25% and a global hypokinesis. The patient was treated with thyroxin at a dose of 150 μg/day. After three months of follow up, TSH was at 4 mUI/l and echocardiography showed a substantial improvement of the left ventricule ejection fraction to 49% (table 1).

## Discussion

Our patient's cases confirmed previous reports concerning the potential reversibility of heart failure after substitutive hormonal treatment [3,4].

Thyroid hormones (TH) interact with the sympathovagal balance but act also on peripheral vascular resistances. The effect of TH on the cardiovascular system would be explained by the transcription's regulation of some genes implicated especially in the contractile system synthesis. More rapid non genomic effects of the TH on the ionic channels of cardiomyocyte's membrane were identified more recently 5. Hypothyroidism results in a bradycardia, a decrease of the myocardial contractility and an increase of the systemic peripheral vascular resistances. A pericardial effusion is the most frequent cardiac manifestation. Hypothyroidism is associated with an increased risk for atherosclerosis and ischemic heart disease and is now considered as proatherosclerosis factor[6,7]. Many of the cardiac manifestations of thyroid dysfunction are associated with alterations in T3-mediated gene expression 8. In hypothyroidism, although cardiac output is reduced, heart failure is relatively rare because there is a lower oxygen demand in the periphery 9. In our two patients, family history was against inherited causes. There were no histories of drug abuse, nor alcohol or toxic substances addiction. Personal history and clinical exam ruled out nutritional deficiency, while negative HIV and HCV serologies eliminated potential viral causes. Our observation highlighted the importance of hormone dosage in first assessment of DCM even in the absence of major symptoms of TH, because clinical presentation is sometimes not obvious and the diagnosis can only be made by thyroid function testing. Fortunately, the diagnosis of hypothyroidism was clinically evident in our two patients and confirmed by hormone study. The second challenge is to know whether TH is an occasional association with DCM or a cause of it [8]. The improvement of the cardiac function after hormonal treatment is an important argument in favor of the implication of hypothyroidism in the genesis of DCM in our patients.

## Conclusion

DCM is usually idiopathic disease with progressive and an irreversible poor prognosis out come. In contrast, in some cases, DCM can be secondary to various causes such as hypothyroidism and hormonal treatment with L thyroxin can significantly improve myocardial function. Hence, thyroid function tests should be systematically performed in all patients with DCM in order to rule out a hypothyroidism.

## Competing interests

The authors declare that they have no competing interests.

## Authors' contributions

IK has written the paper. NH: participated in the design of the study OH: participated in its design and coordination. SH, JS and MG: carried out the cardiac and thyroid imagery. SM: participated in writing the paper. All authors read and approved the final manuscript.

## Consent

Written informed consent was obtained from the patient for publication of this case report and accompanying images. A copy of the written consent is available for review by the Editor-in-Chief of this journal.
